# Fracture Healing Is Delayed in Immunodeficient NOD/scid‑﻿IL2Rγ﻿_c_^null^ Mice

**DOI:** 10.1371/journal.pone.0147465

**Published:** 2016-02-05

**Authors:** Anna E. Rapp, Ronny Bindl, Stefan Recknagel, Annika Erbacher, Ingo Müller, Hubert Schrezenmeier, Christian Ehrnthaller, Florian Gebhard, Anita Ignatius

**Affiliations:** 1 Institute of Orthopaedic Research and Biomechanics, University of Ulm, Ulm, Germany; 2 Department of General Paediatrics, Haematology and Oncology, University Children’s Hospital Tübingen, Tübingen, Germany; 3 Clinic for Paediatric Haematology and Oncology, Bone Marrow Transplantation Unit, University Medical Centre Hamburg-Eppendorf, Hamburg, Germany; 4 Institute of Clinical Transfusion Medicine and Immunogenetics, German Red Cross Blood Transfusion Service and University Hospital Ulm, Baden Wuerttemberg-Hessen, Ulm, Germany; 5 Institute of Transfusion Medicine, University of Ulm, Ulm, Germany; 6 Department of Traumatology, Hand-, Plastic, and Reconstructive Surgery, Centre of Surgery, University of Ulm, Ulm, Germany; Klinikum rechts der Isar - Technical University Munich - TUM, GERMANY

## Abstract

Following bone fracture, the repair process starts with an inflammatory reaction at the fracture site. Fracture healing is disturbed when the initial inflammation is increased or prolonged, whereby, a balanced inflammatory response is anticipated to be crucial for fracture healing, because it may induce down-stream responses leading to tissue repair. However, the impact of the immune response on fracture healing remains poorly understood. Here, we investigated bone healing in NOD/scid-IL2Rγ_c_^null^ mice, which exhibit severe defects in innate and adaptive immunity, by biomechanical testing, histomorphometry and micro-computed tomography. We demonstrated that NOD/scid-IL2Rγ_c_^null^ mice exhibited normal skeletal anatomy and a mild bone phenotype with a slightly reduced bone mass in the trabecular compartment in comparison to immunocompetent Balb/c mice. Fracture healing was impaired in immunodeficient NOD/scid-IL2Rγ_c_^null^ mice. Callus bone content was unaffected during the early healing stage, whereas it was significantly reduced during the later healing period. Concomitantly, the amount of cartilage was significantly increased, indicating delayed endochondral ossification, most likely due to the decreased osteoclast activity observed in cells isolated from NOD/scid-IL2Rγ_c_^null^ mice. Our results suggest that—under aseptic, uncomplicated conditions—the immediate immune response after fracture is non-essential for the initiation of bone formation. However, an intact immune system in general is important for successful bone healing, because endochondral ossification is delayed in immunodeficient NOD/scid-IL2Rγ_c_^null^ mice.

## Introduction

A close relationship exists between the bone and immune systems. Both systems share a large number of regulatory molecules, macrophages and osteoclasts develop from the same progenitor and inflammatory disorders can be associated with bone loss [1–4]. The immune system also appears to play an important role in bone healing, because the fracture repair process starts with an inflammatory response [5, 6]. The fracture leads to tissue damage and blood vessel rupture, initiating acute inflammation and the development of a haematoma, which is characterized by low pH, hypoxia and a high concentration of inflammatory mediators and chemokines being released from resident immune cells after sensing injury associated danger signals [7, 8]. Polymorphonuclear neutrophils (PMNs), which are rapidly recruited at the early stage of inflammation, act against endogenous and exogenous pathogens by secreting reactive oxygen species, proteases and cytokines, and phagocytize debris and dead cell remnants. By releasing chemokines, PMNs attract macrophages, which further remove pathogens and initiate tissue repair by producing pro-angiogenic and trophic factors [9]. Later, the immune response shifts towards adaptive immunity, reflected by the invasion of lymphocytes into the fracture zone [10]. The inflammatory phase is orchestrated by many pro- and anti-inflammatory mediators (e.g. interleukin (IL)-1, IL-6, tumour necrosis factor-α), pro-angiogenic mediators and growth factors (e.g. of the bone morphogenetic protein superfamily) [11]. With the resolution of acute inflammation, mesenchymal progenitor cells are attracted and new bone is formed by intramembranous and endochondral ossification [5, 6].

A balanced inflammation at the fracture site restricts tissue damage and initiates tissue repair by providing pro-angiogenic mediators and attracting mesenchymal progenitors cells, and is, therefore, anticipated to be crucial for fracture healing [6, 12]. In contrast, fracture healing is disturbed when the inflammatory response is increased or prolonged. For example, excessive inflammation associated with complex local tissue injury results in delayed healing [13, 14]. Systemic inflammatory conditions, including polytrauma, which induce an acute systemic immune response, significantly increase the risk for non-union [13, 15]. There is also clinical evidence that fracture healing is disturbed in patients with chronic immune disorders, including rheumatoid arthritis, and diabetes [16, 17].

Currently, it is poorly understood how much inflammation may be too much, and whether an immune response is actually crucial for bone repair. Here, we investigated bone healing in NOD/scid-IL2Rγ_c_^null^ mice, which exhibit severe defects in innate and adaptive immunity, and hypothesized that fracture healing would be considerably disrupted when a balanced immune response, which is proposed to have a positive effect on regeneration, is disturbed.

## Materials and Methods

### Mouse model

All experiments were performed according to national and international regulations for the care and use of laboratory animals and were approved by the Local Ethics Committee (No. 1000,Regierungspräsidium Tübingen, Germany). NOD/scid-Il2Rγ_c_^null^ (NOD.Cg-Prkdc^scid^ Il2Rg^tm1Wjl^/SzJ) and Balb/cByJ (referred to as Balb/c) mice were purchased from Jackson Laboratories (Bar Harbor, ME, USA). The immunedeficient NOD/scid-Il2Rγ_c_^null^ mouse is characterized by the lack of functional monocytes, dendritic cells, natural-killer cells and mature lymphocytes [18]. Furthermore, they display no detectable activity of haemolytic complement. The immunogenic organs are degenerated and consist mainly of stroma. Splenic follicles are absent and lymph nodes are completely atrophic. All other organs are normally developed. The mice exhibit severe deficiencies in cytokine signalling due to the defect in the so-called common gamma-chain of the IL2 receptor (γ_c_). Therefore, the signalling of IL-2, IL-4, IL-7, IL-9, IL-15 and IL-21 is defective [19].

BALB/c mice were chosen as the immunocompetent control, because the spontaneous mutation for scid (severe combined immunodeficiency) in the *Prkdc*-locus (protein kinase, DNA-activated, catalytic polypeptide) originally occurred in this strain.

### Animal studies

To investigate whether the immune defect of NOD/scid-Il2Rγ_c_^null^ mice influences bone formation, the skeleton of Balb/c and NSG (male, 12 weeks old, n = 10 for both genotypes) mice was comparatively analysed as described below. Fracture healing was investigated in NOD/scid-Il2Rγ_c_^null^ and BALB/c mice of the same age (n = 24 per genotype). Surgery was performed as previously described [20]. Briefly, an osteotomy was created at the mid-shaft of the right femur and stabilized using an external fixator that was fitted to the bone using four mini-Schanz screws (axial stiffness 18.1 N/mm, RISystem, Davos, Switzerland). To reduce pain, an analgesic (tramadol hydrochloride, 15 mg/kg) was administered subcutaneously before the operation and via the drinking water (25 mg/L) for the first 3 postoperative days. The mice received daily subcutaneous injections of clindamycin-2-dihydrogenphosphate (45 mg/kg) until the third postoperative day. During the first three post-operative days, the animals were monitored daily, afterwards twice a week until sacrification. Specifically, the use of the operated limb was assessed as well general health condition (bodyweight, grooming, overall behaviour). Eight animals per group were sacrificed by CO_2_ asphyxiation after 21, 28 and 35 days and the osteotomized bone was analysed as described below.

### Biomechanical testing

To determine the mechanical quality of the intact and osteotomized femurs, the flexural rigidity was assessed by a non-destructive three-point bending test using a material testing machine as described previously [20, 21]. Briefly, the proximal end of the femur was fixed to an aluminium cylinder using SelfCem (Heraeus Kulzer, Hanau, Germany). The cylinder was fixed in a hinge joint, serving as the proximal support for the bending test. The femoral condyles rested on the bending support, the distance between both supports being 20 mm (l). The bending load F was applied on the mid-shaft and continuously recorded vs. sample deflection (d) up to a maximum force of 5 N. Because the callus was not always located exactly in the middle of the supports (l/2), the distances between the load vector and the proximal (a) and distal (b) supports were considered when calculating the flexural rigidity EI = k((a^2^b^2^)/3l) [20].

### Micro-computed tomography (μCT)

The intact and osteotomized femurs were scanned using a μCT-device (Skyscan 1172, Bruker, Belgium) at a resolution of 8 μm using a peak voltage of 50 kV and 200 μA. In the cortical bone of the non-osteotomized femurs, a volume of interest (VOI) of 168 μm height was defined in the mid-diaphysis. The trabecular bone was evaluated in the distal part of the femur in a 280 μm high VOI with its lower end 200 μm above the growth plate. To assess the fracture-healing outcome, the former osteotomy gap was defined as the VOI. A global threshold of 37% of the maximal grey value of each specimen was used to distinguish between mineralized and non-mineralized tissue [22]. Common standard parameters of the American Society of Bone and Mineral Research (ASBMR) were evaluated [23, 24].

### Histology and histomorphometry

The osteotomized and intact femora were processed for undecalcified histology. The specimens were fixed in 4% formaldehyde, dehydrated, and embedded in methyl methacrylate. Sections of 10 μm of fractured femurs were stained using Giemsa. To determine the osteoblast numbers, 10-μm thick sections of intact femurs were stained using toluidine blue for better visualization of osteoblasts. To visualize osteoclasts, tartrate resistant acid phosphatase (TRAP) staining was performed using naphthol AS-MX phosphate (Sigma-Aldrich, Taufkirchen, Germany) and Fast Red TR-Salt (Sigma-Aldrich) in 0.2 M acetate buffer pH 5.0. For histomorphometric analysis of the fracture callus, the sections were examined using light microscopy (Leica DMI6000B, Leica, Switzerland) at 50-fold magnification. In the fracture callus, the relative cartilage fraction was analysed using the software Leica MMAF 1.4.0 (MetaMorph^®^, Leica, Switzerland). The osteoblasts and osteoclasts were counted using the OsteoMeasure histomorphometry system (OsteoMetrics, Decatur, USA). The analyses were performed according the recommendations of the ASBMR [23].

### *Ex vivo* analyses of osteoblast- and osteoclast-like cells

To investigate whether osteoblasts and osteoclasts exhibit cell-autonomous defects in the NOD/scid-Il2Rγ_c_^null^ mouse, cell function was analysed *ex vivo*. Osteoblasts were isolated from cortical bone of both Balb/c and NSG mice. Briefly, the bones were minced and digested for 2 h using 300 U/ml collagenase type IV (Sigma-Aldrich) in α-minimum essential eagle medium (α-MEM, Biochrom AG, Berlin, Germany). The bone chips were cultivated in α-MEM supplemented with 15% foetal calf serum (FCS), 4 nM L-glutamine, 100 U/mL penicillin, 0.1 mg/mL streptomycin (all Biochrom AG) and 0.25 mg/mL amphotericin-B (Fungizone^®^, Gibco, Darmstadt, Germany) at 37°C under 5% CO_2_, until colonies started to form. The colonies were sub-cultivated and cells in passages 3 to 5 were seeded for osteogenic differentiation at a density of 10,000 cells/cm^2^ in medium further supplemented with 10 mM disodium β-glycerophosphate and 0.2 mM ascorbate-2-phosphate (all Sigma-Aldrich). After 21 days, matrix mineralization was analysed by von-Kossa-staining. Briefly, incubation with silver nitrate leads to the replacement of calcium with silver ions. Reduction of the silver ions results in a dark staining of mineralized areas. Alkaline phosphatase activity was detected by staining using a commercially available kit (Sigma, Germany).

Osteoclast-like cells (OCL) were generated from bone marrow that was flushed from humeri and tibiae. The cells were plated at 3x10^5^ cells/cm^2^ and cultivated in α-MEM supplemented with 10% FCS (Gibco), 4 nM L-glutamine, 100 U/mL penicillin and 0.1 mg/mL streptomycin (all Biochrom AG). To stimulate osteoclast fusion, 10 ng/mL recombinant human macrophage-colony stimulating factor (rh-MCSF, Chemicon, Limburg, Germany) were added for 3 days. The non-adherent cell fraction was plated at a density of 5x10^5^ cells/cm^2^ on normal tissue culture plastic and in plates with a synthetic calcium phosphate coating (BD BioCoat^™^ Osteologic^™^ Bone Cell Culture System plates; Becton Dickinson GmbH, Germany) and cultivated for 7 days. To assess OCL formation, cells were stained for TRAP using a commercial kit (Sigma, Germany). TRAP-positive cells with ≥3 nuclei were counted as OCL. To analyse the resorption activity of the OCL, BD BioCoat^™^ Osteologic Bone Cell Culture System^™^ slides were treated using 6% sodium hypochloride and von-Kossa stained to visualise the resorbed areas in the calcium phosphate coating.

### Statistics

Data is presented as the mean ± standard error of the mean. Comparisons between two groups of normally distributed variables, analysed using Shapiro-Wilk normality test, were performed using student’s T-test. (GraphPad Prism6, GraphPad Software, Inc., La Jolla, CA, USA). The level of significance was p<0.05.

## Results

### Immunodeficiency in NOD/scid-IL2Rγ_c_^null^ mice induces a moderate bone phenotype

**Fig 1:**
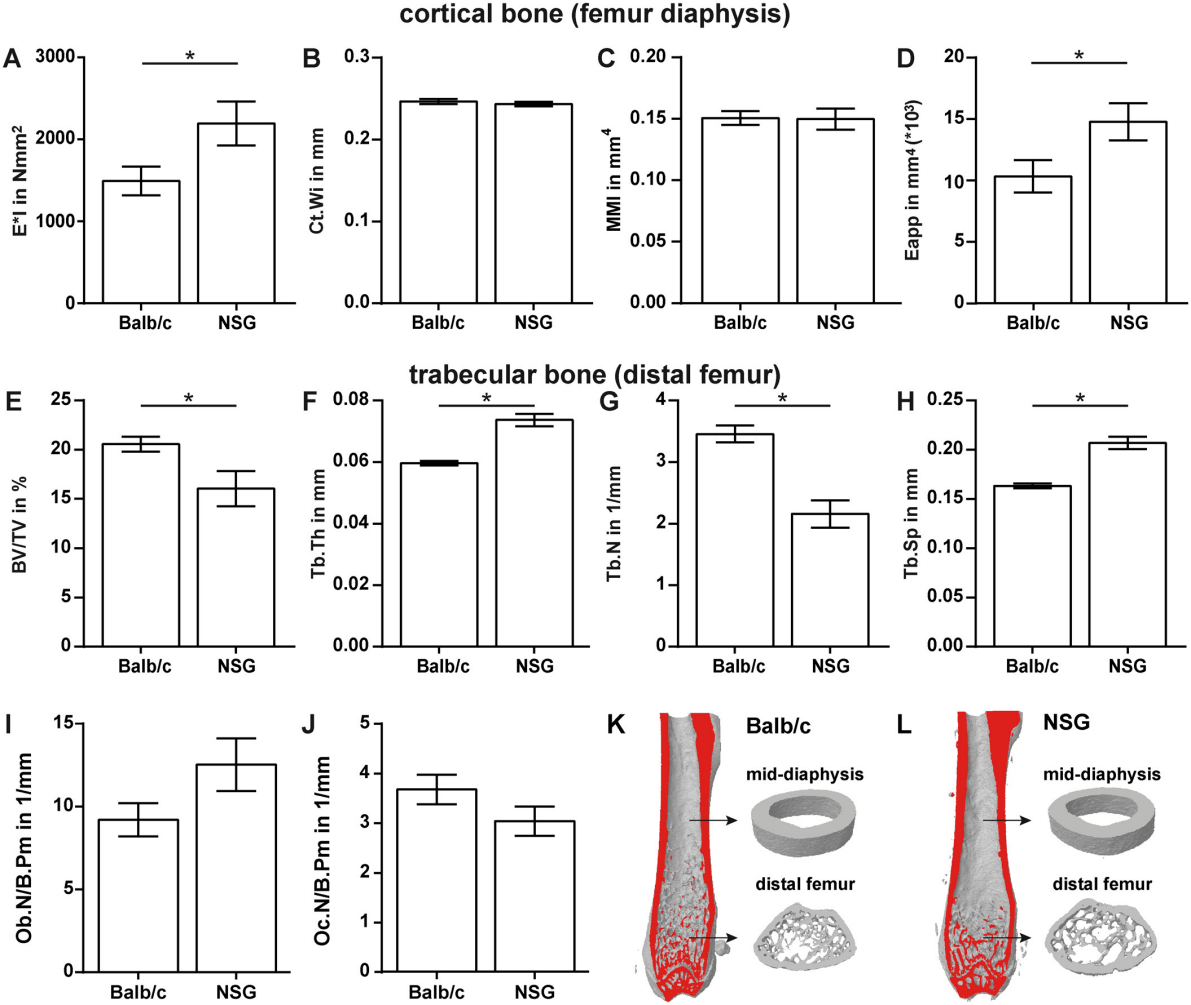
Skeletal phenotype of Balb/c and NOD/scid-IL2Rγc^null^ (NSG) mice. Cortical bone was analysed by a three-point bending test and micro-computed tomography (μCT) (A-D); trabecular bone in the distal femur (E–H) was analysed by μCT. The osteoblast number per bone perimeter (Ob.N/B.Pm) was assessed in toluidine-blue-stained sections (I). The number of osteoclasts per bone perimeter (Oc.N/B.Pm) was evaluated in sections stained for tartrate resistant acid phosphatase (TRAP) (J). K and L depict representative 3-dimensional reconstructions of femurs from Balb/c (K) and NSG mice (L). Data is presented as the mean ± standard error of the mean. n = 9–10. Asterisks denote significant differences; p<0.05.

To assess the skeletal phenotype of NOD/scid-IL2Rγ_c_^null^ mice, whole body X-rays were evaluated in comparison to Balb/c mice. We observed no gross abnormalities when comparing the skeleton of both strains (data not shown). The flexural rigidity of the femurs assessed by three-point bending was significantly higher (+47%, p = 0.0495) in NOD/scid-IL2Rγ_c_^null^ compared to Balb/c mice (Fig 1A). Micro-computed tomography analysis of the femur diaphysis revealed no significant alterations in cortical width (Ct.Wi), moment of inertia (MMI) or mineralization represented by the mean grey value (115.76±1.43 for Balb/c vs. 112.67±1.40 for NOD/scid-IL2Rγ_c_^null^). The apparent Young’s Modulus (Eapp), representing the mechanical properties of the bone matrix, was significantly increased by 43% in NOD/scid-IL2Rγ_c_^null^ (p = 0.0448) (Fig 1B–1D).

Analysis of trabecular bone at the distal femur revealed a moderately reduced bone mass in NOD/scid-IL2Rγ_c_^null^ compared to Balb/c mice (Fig 1E–1H). The bone per tissue volume was significantly decreased due to a reduced trabecular number and increased trabecular spacing, whereas trabecular thickness was increased (all parameters p<0.0001) (Fig 1). Representative 3D-reconstructions of cortical and trabecular bone are depicted in Fig 1K and 1L. Osteoblast and osteoclast numbers were not significantly different (Fig 1I and 1J).

**Fig 2:**
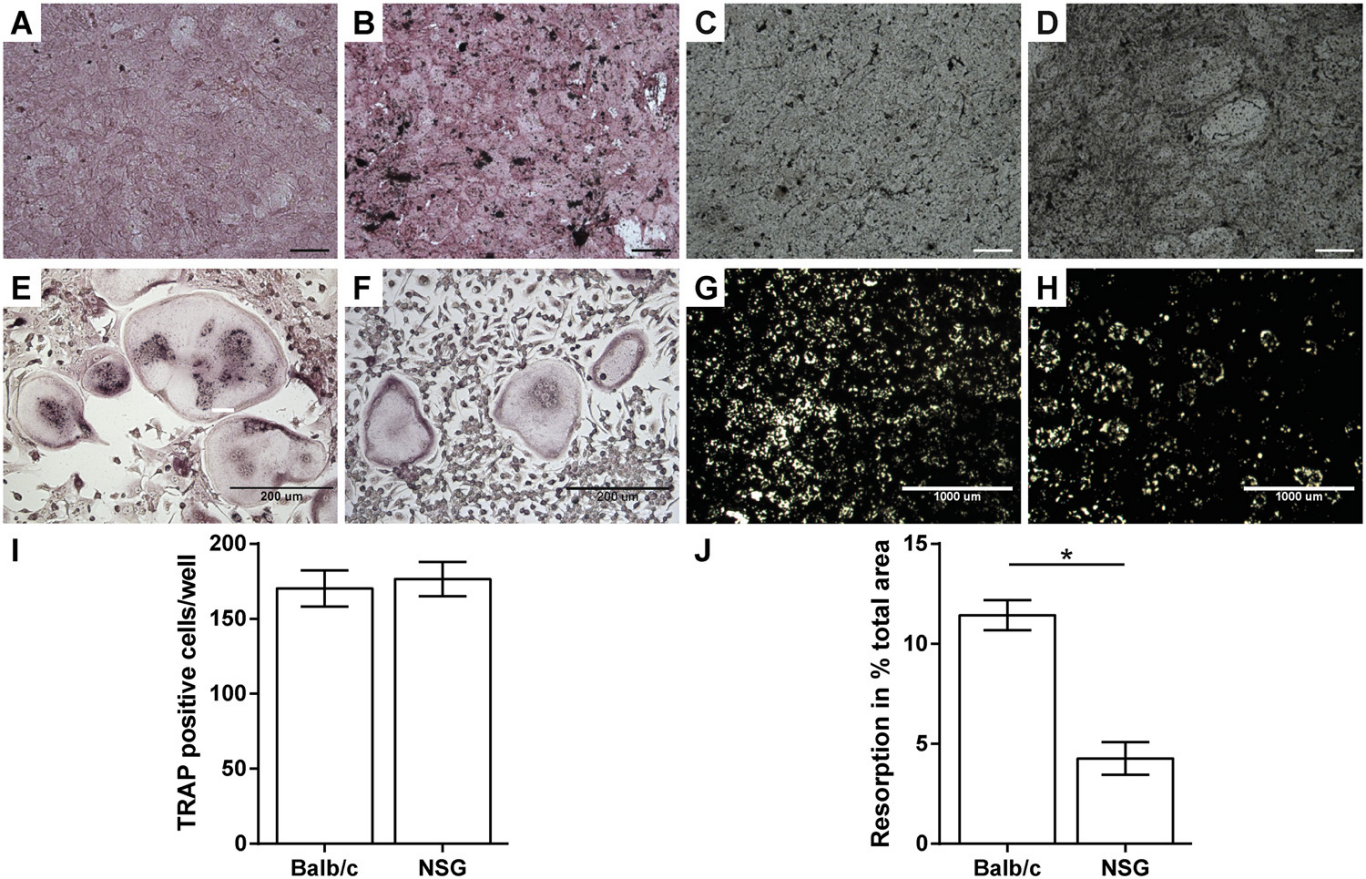
*Ex vivo* analysis of osteoblast and osteoclast function. Osteogenic differentiated primary osteoblasts from Balb/c (A, C) and NOD/scid-IL2Rγ_c_^null^ mice (NSG; B, D) were stained for alkaline phosphatase activity (A, B) and mineral deposition (C, D) using von-Kossa-stain, respectively. Osteoclast-like (OCL) cell fusion from mononuclear cells was analysed using tartrate resistant acid phosphatase (TRAP)-staining (E, F, I). The resorption activity of the OCL was analysed on calcium-phosphate-coated discs. (G, H, J). Data is depicted as the mean ± standard error of the mean. I: n = 4; J: n = 7. Scale bar in A–D = 100 μm.

Primary osteoblasts isolated from NOD/scid-IL2Rγ_c_^null^ mice exhibited a stronger *in vitro* differentiation capacity compared to Balb/c mice, as demonstrated by increased alkaline phosphatase activity and mineral deposition (Fig 2A–2D). The *in vitro* formation of osteoclast-like cells was unaffected in NOD/scid-IL2Rγ_c_^null^ mice (Fig 2E, 2F and 2I); however, the resorption activity of the cells was significantly reduced (Fig 2G, 2H and 2J).

### Immunodeficiency in NOD/scid-IL2Rγ_c_^null^ mice delays fracture healing

**Fig 3:**
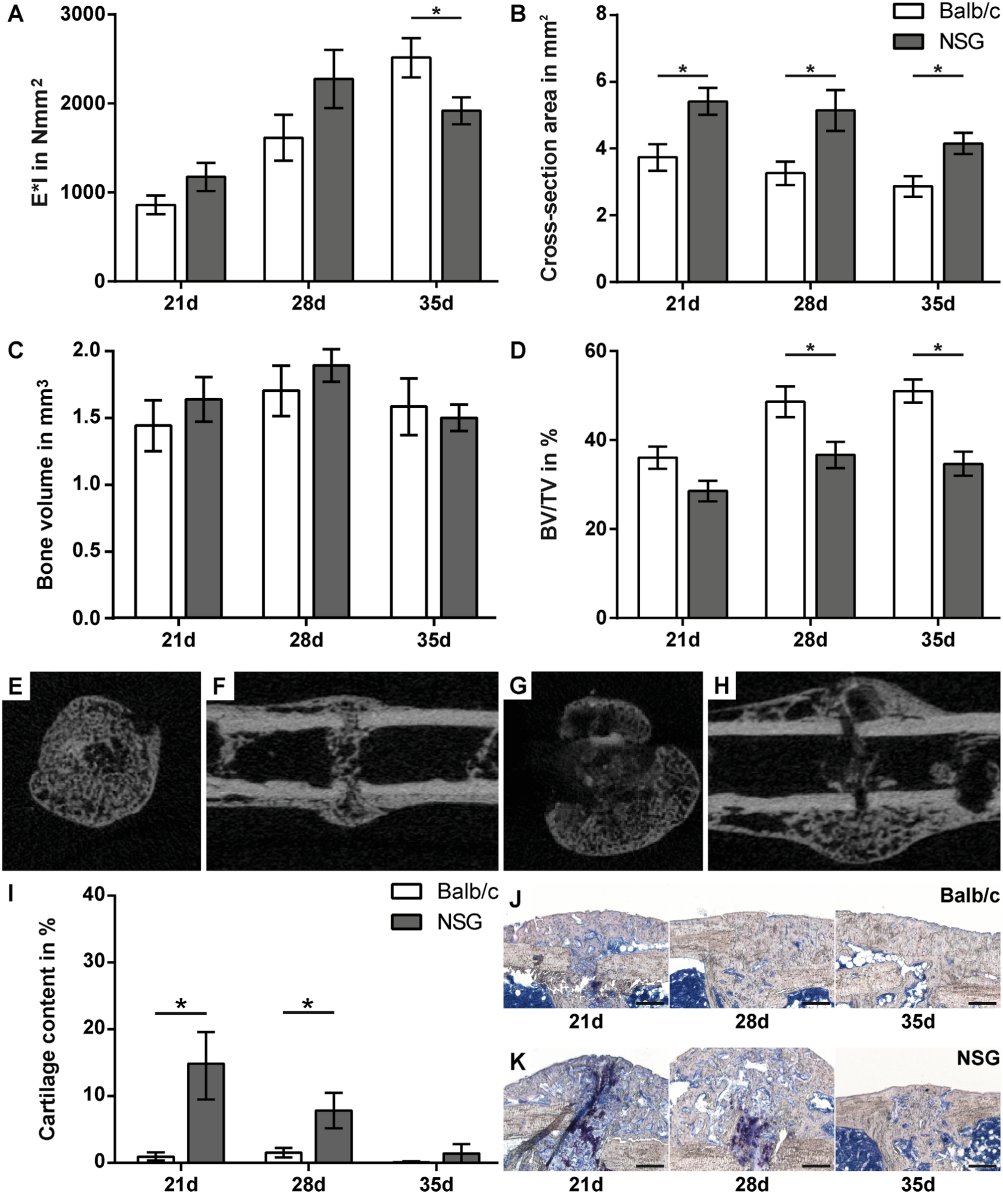
Fracture healing is moderately altered in NOD/scid-IL2Rγc^null^ (NSG) mice. Assessment of the fracture-healing outcome by three-point bending test (A), micro-computed tomography (B-D) and histomorphometry (I). Representative cross sections (E, G) and longitudinal sections (F, H) on day 21 of calli from Balb/c (E, F) and NSG mice (F, G). Representative micrographs of fracture calli of Balb/c (J) and NSG (K) mice on day 21, 28 and 35. Data is presented as the mean ± standard error of the mean; n = 6–8. Asterisks indicate significant differences between Balb/c and NSG mice at the indicated time-point; p<0.05. Scale bars in J and K = 200 μm.

Biomechanical testing of healed femurs in Balb/c and NOD/scid-IL2Rγ_c_^null^ mice revealed no statistically significant differences after a healing period of both, 21 and 28 days. On day 35, flexural rigidity was significantly lower in NOD/scid-IL2Rγ_c_^null^ mice (p = 0.0430) (Fig 3A).

At all time points, μCT analysis demonstrated a significant increase in callus cross-section area in NOD/scid-IL2Rγ_c_^null^ mice compared to Balb/c mice (p = 0.0115–0.015) (Fig 3B and 3E–3H). Although the absolute bone volume was not significantly different (Fig 3C), the relative bone content of the fracture callus was significantly lower in NOD/scid-IL2Rγ_c_^null^ mice on days 28 and 35 (p = 0.0197 and p<0.0006, respectively) due to the increased callus size (Fig 3D).

Histomorphometric analysis revealed a significantly increased cartilage content in the calli of NOD/scid-IL2Rγ_c_^null^ mice on days 21 and 28 (p = 0.048 and 0.0297, respectively) (Fig 3I, 3J and 3K). Therefore, the longer persistence of cartilage and the reduced bone fraction in the facture callus of the immunodeficient mice suggest delayed endochondral bone formation. The reduced callus tissue quality in the NOD/scid-IL2Rγ_c_^null^ mice did not affect the flexural rigidity on days 21 and 28, because the callus was larger. However, on day 35, the greater callus size no longer compensated for the poor quality.

## Discussion

To investigate the importance of the immune response in fracture healing, we studied bone healing in NOD/scid-IL2Rγ_c_^null^ mice, which exhibit severe immune defects. Fracture healing was impaired in the immunodeficient mice compared to immunocompetent Balb/c mice, as demonstrated by a significantly reduced bone content of the fracture callus in the late healing phase. Concomitantly, the amount of cartilage was significantly increased, indicating delayed endochondral ossification.

We chose NOD/scid-IL2Rγ_c_^null^ mice for this study, because they have severe defects in innate and adaptive immunity, but do not exhibit obvious dysfunctions of other organs and have a normal life expectancy. Therefore, these mice are an established model, for example, for xenogeneic transplantation [25]. NOD/scid-IL2Rγ_c_^null^ mice lack functional leucocytes, macrophages, dendritic cells, natural killer cells and lymphocytes. They also display no detectable activity of haemolytic complement. The mice exhibit deficiencies in cytokine signalling due to the lack of the common gamma chain (γ_c_) of the IL-2 receptor [18]. This receptor subunit is described as important, if not essential, for the binding and signalling of various interleukins, including IL-2, IL-4, IL-7, IL-9, IL-15 and IL-21 [26, 27], which also partially play a role in bone homeostasis [28, 29].

Because—to our best knowledge—the bone phenotype was not previously described in detail, we first analysed the skeleton of 12-week-old NOD/scid-IL2Rγ_c_^null^ mice. Gross inspection of the skeleton revealed no obvious abnormalities, indicating that bone development was not disturbed. Furthermore, immunodeficient mice developed only a mild bone phenotype. Structural parameters of the cortical bone were not significantly affected. In contrast, mechanical properties were increased, indicating alterations to the bone matrix that we could not detect by the applied methods. In the trabecular compartment, bone mass was slightly reduced in NOD/scid-IL2Rγ_c_^null^ mice due to a diminished trabecular number; however, the existing bone trabeculae were thicker. Bone cell numbers were unaltered *in vivo*; however, osteoblasts isolated from immunodeficient mice and cultivated *ex vivo* displayed an increased differentiation capacity, whereas osteoclast resorption activity was reduced, indicating cell-autonomous defects. These results explain the increased trabecular thickness in immunodeficient mice. As stated above, cells of the hematopoietic lineage are affected by the defects of the NOD/scid-IL2Rγ_c_^null^ mouse. As osteoclasts derive from the myeloid lineage, the disturbed osteoclast function observed *ex vivo* might be caused by the deletion of the γ_c_-subunit of the IL-2 receptor, which affects the signalling of various interleukins [26, 27]. Cytokine signalling plays a central role in osteoclastogenesis and defects in this signalling pathway could cause osteoclast dysfunctions [30]. The increased differentiation capacity of osteoblasts is difficult to account for by a single underlying factor. Some of the cytokines affected by the γ_c_^null^ mutation also play a role in bone homeostasis [28, 29, 31]; however, the impact on isolated osteoblasts is unclear. In conclusion, 12-weeks-old NOD/scid-IL2Rγ_c_^null^ mice exhibit only a mild bone phenotype in comparison to Balb/c mice.

A possible limitation of our study is that we could not use immunocompetent wild-type mice with the same genetic background as the NOD/scid-IL2Rγ_c_^null^ mice, because these mice were generated by intercrossing several mouse strains with different immune deficiencies [18]. Also, using the founder strains as controls was not possible, as they display abnormalities that NOD/scid-IL2Rγ_c_^null^ mice do not display like wound healing disorders in the NOD/ShiLt mouse. So, as a compromise, we used Balb/c mice as an immunocompetent control because the spontaneous mutation for scid (severe combined immune deficiency) in the *Prkdc*-locus (protein kinase, DNA-activated, catalytic polypeptide) originally occurred in this strain and thus the genetic background may have a high degree of similarity. However, because bone phenotype and healing characteristics in mice depend on the genetic background [32, 33], an influence on bone cell function cannot be completely excluded. To overcome this problem, one could think about a rescue-approach, where bone marrow of immunocompetent mice is transplanted into NOD/scid-IL2Rγ_c_^null^ mice in order to generate a functional immune system. However, irradiation of the mice is necessary for such an approach, thus, other problems arise.

Our results demonstrated that fracture healing was impaired in immunodeficient NOD/scid-IL2Rγ_c_^null^ mice. The callus bone content was unaffected on day 21, but it was significantly reduced in the later healing phase. The longer persistence of cartilage indicates delayed cartilage-to-bone transformation during endochondral ossification, most likely through reduced osteoclast activity. Impaired osteoclast activity is likely to arise from cell-autonomous defects, as suggested by our *ex vivo* experiments, or by the absence of functional immune cells, including T-lymphocytes, which support osteoclast activity by producing receptor activator of nuclear factor κ-B ligand [34]. Reduced osteoclast activity could also account for the increased callus size in NOD/scid-IL2Rγ_c_^null^ mice, which indicates delayed callus remodelling. Osteoclasts are crucial for the degradation of hypertrophic cartilage and for callus remodelling in the later healing period, whereas periosteal primary bone formation during the earlier healing phase is osteoclast independent [35, 36]. Therefore, delayed fracture healing in NOD/scid-IL2Rγ_c_^null^ mice was rather caused by impaired osteoclast function than disturbed osteoblast precursor cell recruitment and differentiation during the early healing period. Therefore, our results suggest that—under sterile, uncomplicated conditions—the immediate immune response after fracture is nonessential for the initiation of bone formation.

Because we aimed to study the general impact of the immune system on fracture healing, we used a mouse model of severe immune deficiency. Other authors investigated the impact of specific immune cell subsets on fracture healing using transgenic mouse models or antibody induced cell depletion [37, 38]. Thereby, the impact of immune cells, which are classically attributed to innate immunity and act primarily as first line of defence, is intensely discussed. Increased recruitment of neutrophils induced by granulocyte-colony stimulating factor was shown to accelerate bone formation [39]. In contrast, neutrophil depletion impaired fracture healing [40]. Therefore, it appears that a balanced activation of PMNs is necessary for regular bone regeneration [41]. Macrophages are generally considered to contribute to the resolution of inflammation and initiation of tissue repair through the clearance of apoptotic neutrophils and secretion of anti-inflammatory factors, including IL-10 and transforming growth factor-β [42]. In support of this, recent data suggest that bone-resident macrophages are necessary for the initiation of fracture repair and promote anabolic mechanisms during intramembranous and endochondral callus formation, as macrophage-depleted mice display delayed healing of bone injuries [9, 43, 44]. We, in contrast, found only minor changes in fracture healing in the macrophage-deficient NOD/scid-IL2Rγ_c_^null^ mouse, possibly because the deficiencies in this model are much more complex.

The role of other innate immune cells, including natural killer cells and mast cells in bone healing remains unclear. Cells attributed to adaptive immunity are believed to play an important role mainly in later stages of fracture healing [10]. Bone healing was improved in lymphocyte-deficient, recombination activating gene-1 (RAG-1)-knockout mice, indicating a negative effect of these cells [37]. In agreement with this, depletion of CD8-positive cells improved fracture healing, whereas transplantation of CD8-positive cells resulted in delayed healing [38]. Furthermore, mice lacking functional B-lymphocytes displayed enhanced bone formation [45]. These studies suggest that lymphocytes may provoke negative effects on bone regeneration. However, the studies mentioned above investigated the effect of single cell types and did not consider the delicate interplay of cells and factors of the immune system.

In conclusion, this study described, for the first time, fracture healing in a mouse model with severe immune deficiency. Fracture healing was delayed due to impaired endochondral ossification. However, primary bone formation in the early healing stage was unaffected in the model used for uncomplicated fracture healing. Further studies are necessary to unravel the multifaceted interactions between immune cells and bone cells in fracture healing.
